# Short and oral antimicrobial therapy for diabetic foot infection: a narrative review of current knowledge

**DOI:** 10.5194/jbji-7-61-2022

**Published:** 2022-03-25

**Authors:** Steven M. Maurer, Zehra S. Hepp, Shawna McCallin, Felix W. A. Waibel, Federico C. Romero, Yılmaz Zorman, Benjamin A. Lipsky, İlker Uçkay

**Affiliations:** 1 Orthopedic Surgery, Balgrist University Hospital, University of Zurich, Zurich, Switzerland; 2 Internal Medicine, Balgrist University Hospital, University of Zurich, Zurich, Switzerland; 3 Clinical and Phage Research, Balgrist University Hospital, University of Zurich, Zurich, Switzerland; 4 Infectiology, Balgrist University Hospital, University of Zurich, Zurich, Switzerland; 5 Department of Infectious Diseases, Sanatorio Allende Hospital, Córdoba, Argentina; 6 Cardiovascular Surgery Department, Koç University Hospital, Istanbul, Turkey; 7 Department of Medicine, University of Washington, Seattle, USA

## Abstract

Diabetic foot infection is a frequent complication in long-standing diabetes mellitus. For antimicrobial therapy of this infection, both the optimal duration and the route of administration are often based more on expert opinion than on published evidence. We reviewed the scientific literature, specifically seeking prospective trials, and aimed at addressing two clinical issues: (1) shortening the currently recommended antibiotic duration and (2) using oral (rather than parenteral) therapy, especially after the patient has undergone debridement and revascularization. We also reviewed some older key articles that are critical to our understanding of the treatment of these infections, particularly with respect to diabetic foot osteomyelitis. Our conclusion is that the maximum duration of antibiotic therapy for osteomyelitis should be no more than to 4–6 weeks and might even be shorter in selected cases. In the future, in addition to conducting randomized trials and propagating national and international guidance, we should also explore innovative strategies, such as intraosseous antibiotic agents and bacteriophages.

## 1 Introduction

Chronic diabetic foot osteomyelitis (DFO) is associated with substantial morbidity, prolonged hospitalizations, and high health care costs (Uçkay et al., 2015, 2018a). The main local causes of DFO are pathological pressure or unperceived microtraumas on a polyneuropathic (and often ischemic) foot, leading to ulcerations that become infected (Pitocco et al., 2019). Thus, the current state-of-the-art management for DFO includes soft tissue (and often bone) debridement, pressure offloading, revascularization, and the administration of antibiotic agents. The optimal duration and route of administration of systemic antibiotic treatment for DFO is largely based on expert opinion, which is supported more by clinical experience than by research evidence. Most textbooks and several local and international guidelines on treating DFO advocate 6 weeks of antibiotic therapy, with at least the initial week or more administered parenterally. As such generalized guidance often does not consider the variations and comorbidities in the affected patients, many regimens are likely more aggressive than needed. In these situations, antibiotic overuse (in spectrum of coverage, duration of treatment, or parenteral administration) is unlikely to lead to a better clinical outcome and, instead, presents the risk of adverse effects, may increase drug-to-drug interactions and the occurrence of *Clostridium difficile* colitis, and certainly increases financial costs (Uçkay et al., 2015; Lipsky et al., 2012; van Asten et al., 2018). In recent years, the results of several comparative trials have suggested that treatment with systemic antibiotic therapy for 3–6 weeks for patients with DFO with unresected infected bone could be sufficient to prevent clinical failures (Uçkay et al., 2015, 2019; Lipsky et al., 2012; Tone et al., 2015) (Table 1).

**Table 1:** Literature randomizing the antibiotic treatment duration for diabetic foot osteomyelitis.

Reference (Country)	Number of episodes	Duration of antibiotics		Minimal follow-up	Major findings
Tone et al. (2015) (France)	40	6 weeks ( n=20 )	12 weeks ( n=20 )	≥12 months after therapy	Overall cure in 26 (65 %) patients; no significant differences between the 6-week and 12-week groups (12 out of 20 patients vs. 14 out of 20 patients, respectively; p=0.50 ) Fewer gastrointestinal adverse events in groups treated for 6 vs. 12 weeks (15 % vs. 45 %, respectively; p=0.04 )
Gariani et al. (2021) (Switzerland)	93	3 weeks( n=44 )	6 weeks( n=49 )	≥2 months after therapy	Cure in 37 (84 %) of patients in the 3-week group vs. 36 (73 %) in the 6-week group ( p=0.21 ; intention-to-treat analysis); 33 out of 39 patients vs. 32 out of 43 patients, respectively; p=0.26 in the per-protocol analysis) Similar occurrence of adverse events (17 out of 44 patients vs. 16 out of 49 patients, respectively; p=0.51 )

**Figure 1:**
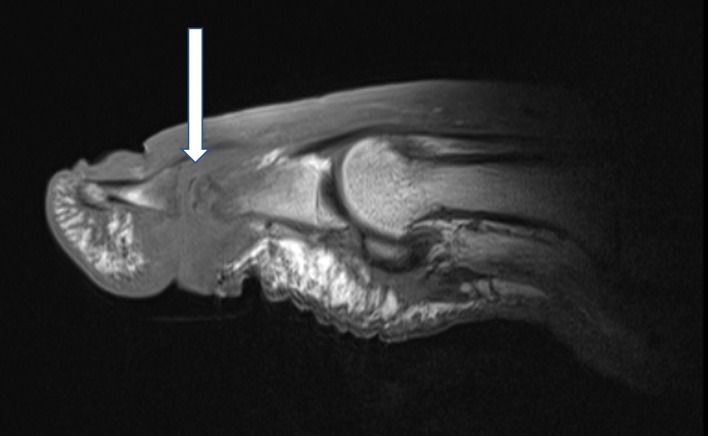
T1-weighted sagittal images of a right first interphalangeal joint demonstrating osteomyelitis of the head of the proximal phalanx and the basis of the distal phalanx. Infectious osteomyelitis is indicated by fat mark suppression in T1 (arrow). Magnetic resonance images with consent of the patient.

**Figure 2:**
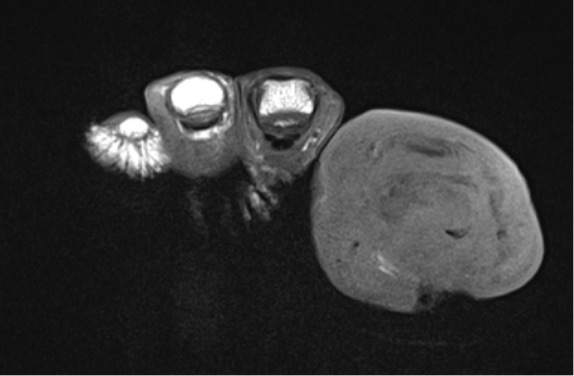
T1-weighted coronal images of the same case as in Fig. 1.

**Figure 3:**
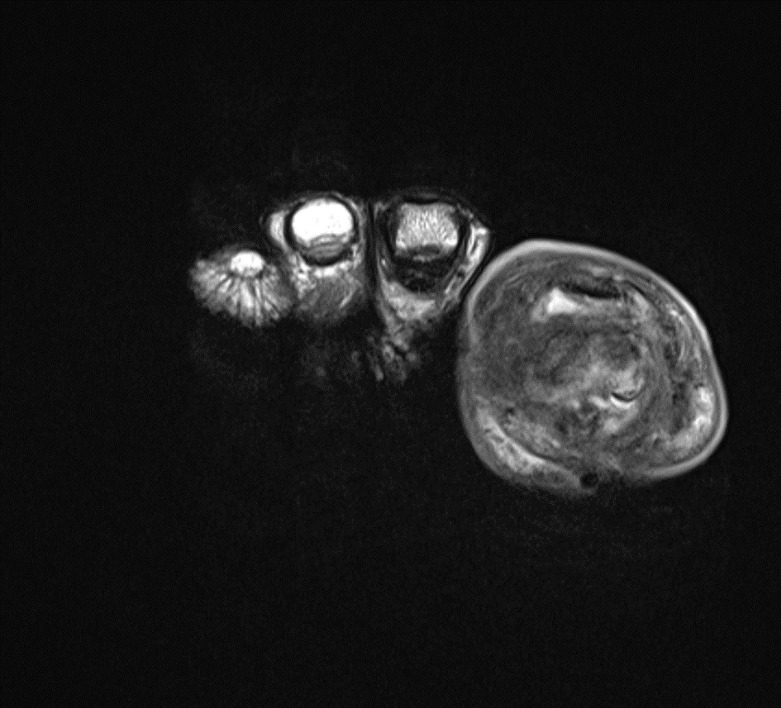
T2-weighted images of the same view as in Fig. 2.

Therefore, we performed a literature search on antibiotic treatment duration and administration routes in DFO patients with a bone infection that was not completely amputated or resected. In this focused review, we will not deal with surgical techniques nor the (radiological) diagnosis of DFO (Figs. 1–3).

## 2 Methods

All of the authors of this paper participated in the literature search using PubMed and Google (Scholar), seeking relevant papers published in the English, German, Spanish, and Turkish languages (based on the individual authors' linguistic skills) any time before 31 December 2021. We sought papers using a combination of the minimal Medical Subject Headings (MeSH) terms “antibiotic treatment”, “duration”, and “diabetic foot osteomyelitis” in these languages. We excluded papers with only in vitro data, without reports of their own patient data, with insufficient results to interpret the results, or with only animal models. The abovementioned authors were specialized in infectious diseases (3 authors), surgery (3 authors), microbiology (1 author), or internal medicine (1 author). We specifically targeted prospective trials investigating the duration and administration routes of antimicrobial therapy for DFO and ignored any other information, such as surgical approaches, epidemiology or diagnosis of DFO, diabetic foot soft tissue infections, or the choice of antimicrobial agents (Abbas et al., 2015). We also did not include data on the management of (acute) DFO in the context of (implant-related) surgical site infections in diabetic patients (Al-Mayahi et al., 2016), which we consider to be a distinct clinical entity.

## 3 Results

### 3.1 Difficulties in conducting prospective trials for DFO

People with DFO are a heterogenous population but usually have advanced peripheral neuropathy and often have peripheral arterial disease (Ertuğrul et al., 2020; Uçkay et al., 2015, 2016; Lipsky, 2021). Evidence from randomized controlled trials offers the highest-quality evidence, but these are difficult to conduct on this complex population. Osteomyelitis must be differentiated from other foot diseases (e.g., ischemic necrosis or Charcot foot deformities) in people with diabetes (Waibel et al., 2022). Moreover, the infected diabetic foot is a dynamic problem that requires the re-evaluation of various confounding features, including during the antimicrobial course. For example, long-duration antibiotic treatment, especially in the presence of an open wound, may lead to the alteration of causative microorganisms during ongoing antibiotic therapy and iterative debridement (Wuarin et al., 2019).

Ideally, every prospective trial regarding DFO and antimicrobial therapy would have two major outcomes. The first is the clinical outcome, either “cure” (curing the symptoms by which the infection was diagnosed) or “failure” (requiring a continued or new therapy for a persistent, recurrent, or new infection). The second is the microbiological outcome, which can be “failure” (the original pathogen(s) is (are) not eradicated) or “recurrence” (the subsequent isolation of a microbiologically identical pathogen at the same location after presumed or proved eradication). The ”microbiological recurrence” would be the only outcome that can be influenced by antimicrobial therapy. Moreover, in a trial assessing the duration of antibiotic therapy, only failures *after* therapy influence the study question. Clinical failures *during* ongoing therapy only assess the performance of a nonsurgical approach per se, not the efficacy of the duration of the administered antimicrobials, as the failure occurred during therapy that was not yet discontinued.

Moreover, almost all prospective controlled trials on (unselected) DFO patients are randomized based on the clinical parameter “infection”, which is not always the most important problem. Another important shortcoming of many studies is the failure to check if the patient has actually taken their antibiotic medication. This concern is bolstered by the notorious difficulty for many diabetic foot patients to comply with pressure offloading as well as the relatively high risk (8 %–15 %) of antibiotic-related adverse events (Gariani et al., 2021). Many studies suggest regularly contacting the patient with reminders, using a patient-filled antibiotic calendar, or requesting that the patient return the empty packages as methods to improve medication compliance (Waibel et al., 2020). Despite the abovementioned shortcomings inherent in many prospective trials in the field of DFO, we think that even the smaller prospective randomized trials are superior to the retrospective studies with respect to providing robust evidence for the optimal duration of antibiotic therapy.

### 3.2 Microorganisms and bone sampling

Any organism on the foot, including relatively low-virulence skin commensals, has the potential to colonize an ulcer and ultimately become a causative pathogen of DFO. As a general rule, the frequency of etiologic pathogens varies depending on where the infection was acquired (community-acquired vs. nosocomial), the patient's geographic location, and probably the duration of the infection (Lipsky and Uçkay, 2021; Uçkay et al., 2014). In monomicrobial DFOs, the predominant pathogens in temperate areas (mainly Europe and North America) are gram-positive cocci, especially staphylococci, streptococci, or enterococci (Lipsky et al., 2006). In DFOs originating from a macerated (or ischemic) ulcer or from (sub)tropical and arid geographical areas (mainly Asia and Africa), gram-negative microorganisms are more common. The anatomic site of infection may also influence the pathogens; for example, calcaneal DFO underlying a macerated ulcer may be associated with a higher likelihood of *Pseudomonas* than DFO affecting a toe (Charles et al., 2015; Waibel et al., 2019; Uçkay et al., 2021). In some patients, especially those with infections associated with a health care institution, drug-resistant pathogens, such as methicillin-resistant *S. aureus* (MRSA) (Zenelaj et al., 2014) are (co-) pathogens of DFO. Gariani et al. (2019b) found that neither MRSA nor obligate anaerobes were associated with worse outcomes than other microorganisms. Other studies have similarly found no evidence that any specific pathogen was associated with an increased risk of DFO recurrence (Zenelaj et al., 2014; Charles et al., 2015).

### 3.3 Choice of antimicrobial agents

To date, there are no studies demonstrating the superiority of any one or combination of systemic antibiotic agents over others, either in terms of clinical cure of infection or healing time in ulcerated DFOs (Uçkay et al., 2015; Kruszewska et al., 2021; Abbas et al., 2015; Selva Olid et al., 2015). In published studies, the most frequently used agents in clinical trials have been penicillins, cephalosporins, carbapenems, metronidazole, clindamycin, linezolid, daptomycin quinolones, and vancomycin (Gariani et al., 2021). One agent that might be associated with better outcomes in chronic (staphylococcal) DFO with a substantial quantity of biofilms is rifampin, which must always be used in combination with another anti-staphylococcal agent (Wilson et al., 2019) that must also be microbiologically active against the staphylococci causing the infection. Among the earliest studies was a non-comparative observational study of 17 DFO patients treated with ofloxacin–rifampicin, which achieved a cure in 88 % of patients (Senneville et al., 2001). Given the potential problem of drug interactions with rifampin, prospective randomized trials of the possible benefit of adding rifampin in DFO therapy are required; one such trial is currently under way in the US Veterans Administration system (Bessesen et al., 2020).

### 3.4 Empirical antibiotic therapy

For most community-acquired DFO episodes, no empirical antibiotic therapy is required. As DFO is not an emergency (in contrast with many soft tissue infections), the antibiotic treatment of DFO can be (in most or even all cases) based on the results of bone biopsies, if feasible, as recommended by many experts. However, in a few circumstances (e.g., negative cultures, patient refusal, prior antibiotic therapy, or laboratory flaws), we still may need an empirical choice based on local epidemiology and clinical experience. For community-acquired infections, empirical aminopenicillins and cephalosporins are the antibiotic agents most frequently prescribed for cases classified as mild or moderate (Selva Olid et al., 2015; Gariani et al., 2021). Severe infections require a broader spectrum, at least until the causative pathogens and their susceptibilities are defined. Many clinicians also broaden the spectrum of the empiric regimen when treating a clinical recurrence following apparently successful therapy of a prior infection. This approach is based on the theoretical concern that the previously sensitive pathogens may have developed resistance during treatment, but it does not reflect proven clinical experience. Indeed, we have found that pathogens isolated in iterative DFO episodes at the same location are no more likely to be antibiotic-resistant than in the prior episodes. In two-thirds of clinical recurrences, the pathogens were different from those that caused the earlier episodes (Lebowitz et al., 2017). These data suggest that there is no necessity to broaden the empiric antibiotic spectrum when treating recurrent DFO. Similarly, colonization of the patient with (health-care-associated) MRSA or extended-spectrum β -lactamase-carrying gram-negative rods in other parts of the body (Agostinho et al., 2013) rarely requires an anti-MRSA coverage in hemodynamically stable patients (Zenelaj et al., 2014; Charles et al., 2015).

### 3.5 Administration routes

The most recent guidance from the International Working Group on the Diabetic Foot (IWGDF) recommends that most mild and many moderate infections may be treated by oral agents from the start, whereas severe infections should be treated with intravenous agents, with conversion to oral therapy as soon as the patients clinically improve (Lipsky et al., 2020). Several studies have shown no difference in cure rates between DFO patients who initially (or predominantly) received intravenous compared with oral antibiotic therapy, including treatment with β -lactam antibiotics (Gariani et al., 2019b; Lázaro Martínez et al., 2019). Although aminopenicillin and β -lactam antibiotics have generally been found to lack good bone penetration in vitro studies, this does not seem to matter in daily clinical care, particularly when the infected bone has been debrided or partially amputated. In one of our single-center cohort studies, treatment with oral β -lactam therapy (with oral co-amoxiclav in more than 90 % of cases) did not influence the cure of DFO compared with other antibiotic agents (Gariani et al., 2019a). A prospective randomized trial in DFO patients comparing antibiotic treatment (without surgical resection of bone) for 90 d with surgery plus antibiotic therapy (with oral antibiotics given very early in the course) found that the outcomes were equivalent (Lázaro-Martínez et al., 2014). Today, 90 d is beyond the recommended duration for the antibiotic treatment of DFO, which was not diagnosed in this study on the basis of a bone sample culture. A retrospective study found similar outcomes in DFO episodes treated with more than 1 week of intravenous antibiotics compared with patients receiving less than 1 week of treatment (Gariani et al., 2019a). A British randomized controlled study of the treatment of musculoskeletal infections (the OVIVA trial, which included a subset with DFO) demonstrated the non-inferiority of oral antibiotic therapy during the first 6 weeks (after 1 week of parenteral administration) compared with an intravenous regimen throughout. As expected, the risk of intravenous catheter-associated complications and the financial cost were lower in the patients randomized to oral therapy (Li et al., 2015). Unfortunately, there is a persistent reluctance among many physicians to select oral antibiotic regimens for DFO, largely based on concerns of hampered clinical efficacy related to concomitant arteriopathy that may limit the delivery of the drug to the foot or low serum levels that may impair bone penetration of the drug. There are, however, no published data to support these fears (Uçkay et al., 2019).

### 3.6 Intraosseous and topical antimicrobials

Topical antimicrobial agents might be useful in superficial ulcer infections, but they have no role (at least on their own) in the treatment of deep DFO. Topical therapy should not be confused with a surgically inserted, intraosseous antimicrobial therapy, sometimes referred to as *local* therapy. These latter agents, similar to antibiotic-loaded space-fillers, might be an adjunct option for treating larger bones in the diabetic mid- and hind-foot. Many research groups advocate this approach, which has several theoretical advantages over systemic therapy: they may be useful for patients who are unable to take oral antibiotic pills, and they have the additional ability to fill dead space in the bone. Although local antibiotic treatments are widely used for DFO, there is little high-quality evidence on the appropriate indications, best techniques, proper dosages, elution properties, or pharmacokinetics (Lipsky and Uçkay, 2021). The largest published report is a retrospective review of patients with forefoot DFO who did or did not have perioperative antibiotic-impregnated calcium sulfate implanted (Qin et al., 2019). The authors found that the antimicrobial implants did not improve the rate of (or shorten the time to) healing nor reduce the postoperative amputation rate. The did, however, reduce recurrences of DFO, although at the price of about one-third of the patients having wound leakage lasting for a couple of weeks (Qin et al., 2019). In most publications, intraosseous antimicrobial therapy was administered concomitantly with systemic antibiotic agents and generally showed no additional benefit over systemic therapy alone with respect to the rate of clinical or microbiological cure (Chatzipapas et al., 2020). We encourage research clinicians to investigate the role of the potential systemic antibiotic sparing effect of local intraosseous therapies, at least for relatively larger foot bones, such as the calcaneus or the talus.

### 3.7 Total duration of (post-debridement) systemic antimicrobial treatment

The optimal duration of systemic antimicrobial treatment in DFO, especially after a surgical debridement or partial amputation, remains unclear and is a topic of current research (Table 1). Although some clinicians treat DFO for months, recommendations based on a systematic review of the literature and the current guidelines of IWGDF are a treatment duration of 4–6 weeks (Lipsky et al., 2020). A small randomized controlled trial comparing the outcomes of antibiotic therapy (without surgery) for 6 weeks vs. 12 weeks found a similar rate of clinical cure. Based largely on this landmark study, most clinicians treat DFO for a maximal duration of 6 weeks. However, even shorter durations of antibiotic therapy might be sufficient if there has been a debridement to remove (at least partially) most of the infected and/or necrotic bone. Our prospective randomized non-inferiority pilot trial showed that 3 weeks of antibiotic therapy was not inferior to 6 weeks of therapy in cases of post-debridement DFO (Gariani et al., 2021), albeit with a wide statistical margin of 25 %. Overall, the rate of clinical cure at 2 months was 78 %, which is similar to that reported in other published DFO series. The risk of microbiological recurrences was lower than that of overall clinical failures (Gariani et al., 2021). A large confirmatory trial to test these latter findings (which is currently under way in Zurich) has not detected a major difference in overall outcomes when comparing 3 weeks and 6 weeks post-debridement for DFO in interim evaluations (Waibel et al., 2020). This trial also includes DFO episodes without surgical debridement, in which the wound has been only debrided by specialized nurses.

Importantly, all of these trials presume the existence of a minimal threshold, below which a shorter course of treatment would lead to significantly more failures. This remains a presumption. Available retrospective analyses have regularly failed to yield a minimal duration for antimicrobial therapy for DFO patients (Gariani et al., 2019b). It might be that bone infections are generally so different from one episode (patient) to another that we cannot generalize and have to treat each case individually, analogous to soft tissue infections. It is also unclear how one should consider the role of antimicrobial therapy that was administered prior to surgery or debridement. Most experts begin recording the duration of antimicrobial therapy from the date of complete surgical debridement, presuming that prior antibiotic treatment does not count, but this is again a presumption that requires confirmation by prospective studies. Of note, in all of our own retrospective analyses, administering presurgical antibiotic therapy did not appear to influence the final outcomes of DFO treatment (Gariani et al., 2019b).

### 3.8 Duration of antibiotic treatment after amputation for DFO

A recent area of interest has been the role of residual bone culture after surgical resection in determining the need for further antibiotic therapy. Specifically, the following question has been raised: is it useful to obtain routine microbiological or histological assessment of the residual, proximal bone stump to see if there is still infection present after presumed complete resection of infected bone in DFO? Kowalski et al. (2011) demonstrated that, among DFO patients who had undergone bone resection, patients with a positive culture or histological evidence of infection of the marginal bone had a higher rate of re-amputations than those without (44 % vs. 15 %, respectively). Atway et al. (2012) reported a 41 % incidence of positive bone resection being associated with a worse outcome, despite 25 d of post-amputation antibiotic therapy.

Some data suggest that if the surgeon is confident based on the gross appearance at surgery that all infected bone has been resected, there may be no need to continue the antimicrobial therapy. In DFO cases, Rossel et al. (2019) found that there was no difference in outcome when antibiotics were either stopped immediately after amputation or continued for more prolonged therapy. Likewise, Aragón-Sánchez et al. (2021) performed a retrospective study to address the hypothesis that DFO recurrence is not clinically associated with culture-positive bone margins nor a positive histology. After surgery, antibiotics were immediately stopped in 19 (68 %) patients and continued in 9 (32 %) patients for a median period of 4 d. Despite the fact that the microbiology was positive in 20 (71 %) cases and the histology was positive in 7 (25 %) episodes, they detected a recurrence of DFO in only 3 (11 %) patients; 17 patients (68 %) with microbiological-positive margins and six (24 %) patients with histology-positive margins did not have a recurrence of infection (Aragón-Sánchez et al., 2021). This suggests that osseous proximal stump margins are likely to be contaminated during (or possibly after) collection in surgery. Mijuskovic et al. (2018) suggested that the assessment of residual bone infection should perhaps not rely solely on culture results. Positive cultures without concomitant histological confirmation might overestimate the true rate of residual osteomyelitis. Senneville et al. (2020) suggested that 1–3 weeks of antimicrobial therapy after bone resection should be sufficient if all visibly infected bone has been removed. Other studies advocate that 5 d of post-surgical antibiotic continuation is sufficient for any potential residual bone infection after resection (Saltoglu et al., 2015), whereas the IWGDF recommends 4–6 weeks (Lipsky et al., 2020). The authors of this paper are currently conducting a trial randomizing “unexpected” residual DFO after amputation with 1 vs. 3 weeks of antimicrobial therapy (Waibel et al., 2020).

### 3.9 Antibiotic stewardship and clinical pathways

DFOs are probably among the most frequent reasons for antibiotic overuse worldwide (Uçkay et al., 2019). We think that adhering to the principles of antibiotic stewardship can improve this situation. The most effective measures relating to antibiotic stewardship are making a correct diagnosis, prescribing an antibiotic regimen with the narrowest effective spectrum, and limiting the duration of antibiotic treatment. Putting these principles into practice requires an individual commitment and the courage (on the part of clinicians) to change habitual prescription patterns. In addition, effective surgical draining and resecting of infected and necrotic material can improve the treatment outcome (Uçkay et al., 2019).

Clinical pathways and multidisciplinary teams for managing DFOs have been instituted in some medical centers; however, they also have their limitations: (1) it is difficult to find a universally agreeable time to bring the various team members together, (2) the number of patients requiring evaluation often exceeds the capacity of fixed regular meetings, and (3) the meetings are time-consuming and busy key members may be absent. Implementing order sets (especially if they are embedded within interactive electronic websites) (Uçkay et al., 2014) can be an effective tool to implement “bundles” of approaches and, hopefully, may reduce the antibiotic duration in the management of infection. The academic experience of order sets must be further evaluated in the field of DFO. There are also many administrative approaches that might improve antibiotic stewardship in DFO. Governments can take the lead in initiating diabetic foot centers (Cawich et al., 2014) or organizing regular workshops and public educational lectures. Ensuring development and access to regional (Peter-Riesch et al., 2021) or international guidelines must also be encouraged.

### 3.10 Antibiotic-related side effects during DFO therapies

Drug-related adverse effects are frequent in patients treated with long-lasting antimicrobial regimens. Based on prospective trials on the infected diabetic foot, the incidence of adverse effects ranges from 8 % to 15 % (Gariani et al., 2019b; Uçkay et al., 2018b). These events mostly evolve during the first 3 weeks of antibiotic therapy, and the risk depends on the specific treatment agent. In the study by Tone et al. (2015), patients in the 12-week antibiotic group had a 50 % adverse-event frequency, compared with only 30 % of those in the 6-week group ( p=0.04 ). The most commonly diagnosed events in the 12-week group were hepatic cholestasis (15 %), diarrhea (10 %), vomiting (10 %), and nausea (10 %) (Tone et al., 2015). Another important adverse effect of any long-lasting antibiotic treatment that all clinicians should be concerned about is the potential to induce antibiotic resistance. The proportion of DFO caused by multiresistant microorganisms is probably increasing worldwide (Lipsky, 2016). Van Asten et al. (2018) showed an acquired resistance rate of 14.6 % among all DFO patients within 1 year of diagnosis.

### 3.11 Bacteriophages for DFO

Given the high frequency of DFO and the suboptimal outcomes of antibiotic therapy, there is great interest in finding alternative antimicrobial strategies. One strategy that has recently engendered much interest is bacteriophage (phage) therapy, i.e., the use of natural, lytic viruses of bacteria for the treatment of bacterial infections. These have been used for decades for many types of infections, including various wounds, and more recently for DFO (Fish et al., 2016). Certain properties inherent of phages, such as their anti-biofilm activity, high specificity to target pathogen, low risk of side effects, and ability to self-amplify at the site of infection, make them attractive for further development. At the same time, due to the extremely narrow spectrum of phages, when dealing with polymicrobial infections, treatment will rely on the “head of the snake” paradigm, i.e., predominantly targeting the most abundant pathogen(s) (Joseph and Lipsky, 2010). As with antibiotic therapy, proper sampling to identify causative pathogens is essential for selecting the appropriate phages. For the treatment of infected wounds, the topical route of administration is mostly widely used, facilitating the delivery of phages to the site of infection (Genevière et al., 2021; Duplessis and Biswas, 2020). The utility of the systemic (usually intravenous) application of either phages or concomitant antibiotics in additional to local application in DFO treatment is not clear, especially for the primary site of infection. The optimal frequency of administration, as well as length of treatment, is difficult to ascertain from the available literature, with treatments ranging from daily or alternating days to weekly applications for as long as 2 to 18 weeks (Genevière et al., 2021; Duplessis and Biswas, 2020).

In terms of clinical benefit, studies of phage treatment of DFO have reported clinical resolution for nine patients with topical (and in one case additional oral) administration (Fish et al., 2016, 2018a, b; Nadareishvili et al., 2020). In a prospective study of chronic nonhealing wounds where over half of participants had diabetes, successful outcomes were reported for 74 % of diabetic patients compared with 91 % of non-diabetic patients (Patel et al., 2021). All reported patients had previously undergone failed conventional antibiotic therapy, suggesting that phage therapy provided a benefit for the individual patients. There are few mentions of a local reaction with topical phage applications (e.g., redness or irritation), but the minimal reports of adverse events generally support the safety of this approach. This is almost entirely due to the purity of the phage product applied and will not be a concern for manufactured phages. However, the quality of this evidence remains low. We are aware of two randomized placebo-controlled clinical trials that are currently recruiting: one employing the topical administration of a static composition of phages against *S. aureus*, *P. aeruginosa*, and/or *A. baumannii* (NCT04803708), and another using topical phages alone or with additional intravenous application of personalized phages for *S. aureus* DFO (NCT05177107). Both studies appear to be using phages as an adjunct therapy, allowing for concomitant antibiotic therapy, while the first applies phages only topically. Phage therapy could only progress to become a viable treatment option for DFO through well-structured clinical trials.

## 4 Discussion and conclusions

Antimicrobial therapy is one of the cornerstones in the management of DFO, especially in patients who do not undergo complete bone resection. There now are strong data suggesting that an early switch to oral therapy is usually safe and effective. This allows for the use of more convenient and less costly oral antibiotics very early in the course of treatment, maybe even from the start, for select stable patients (Embil et al., 2006). We hope that this narrative review will help persuade clinicians who treat these difficult infections that the maximum duration of antibiotic therapy should be no more than to 4–6 weeks and that even shorter durations might be possible in select cases. In addition to conducting classical randomized trials and propagating established national and international guidance, we should also further explore innovative antimicrobial strategies, such as intraosseous antibiotic agents for non-resected, large bone infections and targeted bacteriophages. We have made great progress in treating DFO over the past decade, but there is still a long way to go.

## Data Availability

No data sets were used in this article.

## References

[bib1.bib1] Abbas M, Uçkay I, Lipsky BA (2015). In diabetic foot infections antibiotics are to treat infection, not to heal wounds. Expert Opin Pharmacother.

[bib1.bib2] Agostinho A, Renzi G, Haustein T, Jourdan G, Bonfillon C, Rougemont M, Hoffmeyer P, Harbarth S, Uçkay I (2013). Epidemiology and acquisition of extended-spectrum beta-lactamaseproducing Enterobacteriaceae in a septic orthopedic ward. Springerplus.

[bib1.bib3] Al-Mayahi M, Cian A, Kressmann B, de Kalbermatten B, Rohner P, Egloff M, Jafaar J, Malacarne S, Miozzari HH, Uçkay I (2016). Associations of diabetes mellitus with orthopaedic infections. Infect Dis (Lond).

[bib1.bib4] Aragón-Sánchez J, Víquez-Molina G, López-Valverde ME (2021). Controversial Issues Regarding Positive Bone Margins in Surgery for Diabetic Foot Osteomyelitis: A Pilot Study. Int J Low Extrem Wounds.

[bib1.bib5] Atway S, Nerone VS, Springer KD, Woodruff DM (2012). Rate of residual osteomyelitis after partial foot amputation in diabetic patients: a standardized method for evaluating bone margins with intraoperative culture. J Foot Ankle Surg.

[bib1.bib6] Bessesen MT, Doros G, Henrie AM, Harrington KM, Hermos JA, Bonomo RA, Ferguson RE, Huang GD, Brown ST (2020). A multicenter randomized placebo controlled trial of rifampin to reduce pedal amputations for osteomyelitis in veterans with diabetes (VA INTREPID). BMC Infect Dis.

[bib1.bib7] Cawich SO, Islam S, Hariharan S, Harnarayan P, Budhooram S, Ramsewak S, Naraynsingh V (2014). The economic impact of hospitalization for diabetic foot infections in a Caribbean nation. Perm J.

[bib1.bib8] Charles PG, Uçkay I, Kressmann B, Emonet S, Lipsky BA (2015). The role of anaerobes in diabetic foot infections. Anaerobe.

[bib1.bib9] Chatzipapas C, Kougioumtzis IE, Karaglani M, Panagopoulos P, Panopoulou M, Papazoglou D, Drosos GI, Papanas N (2020). Local Antibiotic Delivery Systems in the Surgical Treatment of Diabetic Foot Osteomyelitis: Again, No Benefit?. Int J Low Extrem Wounds.

[bib1.bib10] Duplessis CA, Biswas B (2020). A Review of Topical Phage Therapy for Chronically Infected Wounds and Preparations for a Randomized Adaptive Clinical Trial Evaluating Topical Phage Therapy in Chronically Infected Diabetic Foot Ulcers. Antibiotics (Basel).

[bib1.bib11] Embil JM, Rose G, Trepman E, Math MC, Duerksen F, Simonsen JN, Nicolle LE (2006). Oral antimicrobial therapy for diabetic foot osteomyelitis. Foot Ankle Int.

[bib1.bib12] Ertuğrul B, Uçkay I, Schöni M, Peter-Riesch B, Lipsky BA (2020). Management of diabetic foot infections in the light of recent literature and new international guidelines. Expert Rev Anti Infect Ther.

[bib1.bib13] Fish R, Kutter E, Wheat G, Blasdel B, Kutateladze M, Kuhl S (2016). Bacteriophage treatment of intransigent diabetic toe ulcers: a case series. J Wound Care.

[bib1.bib14] Fish R, Kutter E, Bryan D, Wheat G, Kuhl S (2018). Resolving Digital Staphylococcal Osteomyelitis Using Bacteriophage-A Case Report. Antibiotics (Basel).

[bib1.bib15] Fish R, Kutter E, Wheat G, Blasdel B, Kutateladze M, Kuhl S (2018). Compassionate Use of Bacteriophage Therapy for Foot Ulcer Treatment as an Effective Step for Moving Toward Clinical Trials. Methods Mol Biol.

[bib1.bib16] Gariani K, Lebowitz D, Kressmann B, von Dach E, Sendi P, Waibel F, Berli M, Huber T, Lipsky BA, Uçkay I (2019). Oral amoxicillin-clavulanate for treating diabetic foot infections. Diabetes Obes Metab.

[bib1.bib17] Gariani K, Lebowitz D, von Dach E, Kressmann B, Lipsky BA, Uçkay I (2019). Remission in diabetic foot infections: Duration of antibiotic therapy and other possible associated factors. Diabetes Obes Metab.

[bib1.bib18] Gariani K, Pham TT, Kressmann B, Jornayvaz FR, Gastaldi G, Stafylakis D, Philippe J, Lipsky BA, Uçkay L (2021). Three Weeks Versus Six Weeks of Antibiotic Therapy for Diabetic Foot Osteomyelitis: A Prospective, Randomized, Noninferiority Pilot Trial. Clin Infect Dis.

[bib1.bib19] Genevière J, McCallin S, Huttner A, Pham TT, Suva D (2021). A systematic review of phage therapy applied to bone and joint infections: an analysis of success rates, treatment modalities and safety. EFORT Open Rev.

[bib1.bib20] Joseph WS, Lipsky BA (2010). Medical therapy of diabetic foot infections. J Vasc Surg.

[bib1.bib21] Kowalski TJ, Matsuda M, Sorenson MD, Gundrum JD, Agger WA (2011). The effect of residual osteomyelitis at the resection margin in patients with surgically treated diabetic foot infection. J Foot Ankle Surg.

[bib1.bib22] Kruszewska K, Wesolowska-Gorniak K, Czarkowska-Paczek B (2021). A Comparative Analysis of Antibiotic Usage in Diabetic Foot Infections Against Healing Time. J Foot Ankle Surg.

[bib1.bib23] Lázaro-Martínez JL, Aragón-Sánchez J, García-Morales E (2014). Antibiotics versus conservative surgery for treating diabetic foot osteomyelitis: a randomized comparative trial. Diabetes Care.

[bib1.bib24] Lázaro Martínez JL, García Álvarez Y, Tardáguila-García A, García Morales E (2019). Optimal management of diabetic foot osteomyelitis: challenges and solutions. Diabetes Metab Syndr Obes.

[bib1.bib25] Lebowitz D, Gariani K, Kressmann B, Dach EV, Huttner B, Bartolone P, Lê N, Mohamad M, Lipsky BA, Uçkay I (2017). Are antibiotic-resistant pathogens more common in subsequent episodes of diabetic foot infection?. Int J Infect Dis.

[bib1.bib26] Li HK, Scarborough M, Zambellas R, Cooper C, Rombach I, Walker AS, Lipsky BA, Briggs A, Seaton A, Atkins B, Woodhouse A, Berendt A, Byren I, Angus B, Pandit H, Stubbs D, McNally M, Thwaites G, Bejon P (2015). Oral versus intravenous antibiotic treatment for bone and joint infections (OVIVA): study protocol for a randomised controlled trial. Trials.

[bib1.bib27] Lipsky BA (2016). Diabetic foot infections: Current treatment and delaying the `post-antibiotic era'. Diabetes Metab Res Rev.

[bib1.bib28] Lipsky BA, Christopher E, Attinger J, Steinberg S (2021). Functional Limb Salvage: The Multidisciplinary Approach.

[bib1.bib29] Lipsky BA, Uçkay İ (2021). Treating Diabetic Foot Osteomyelitis: A Practical State-of-the-Art Update. Medicina (Kaunas).

[bib1.bib30] Lipsky BA, Berendt AR, Deery HG, Embil JM, Joseph WS, Karchmer AW, LeFrock JL, Lew DP, Mader JT, Norden C, Tan JS (2006). Diagnosis and treatment of diabetic foot infections. Plast Reconstr Surg.

[bib1.bib31] Lipsky BA, Berendt AR, Cornia PB, Pile JC, Peters EJ, Armstrong DG, Deery HG, Embil JM, Joseph WS, Karchmer AW, Pinzur MS, Senneville E (2012). Infectious Diseases Society of America clinical practice guideline for the diagnosis and treatment of diabetic foot infections. Clin Infect Dis.

[bib1.bib32] Lipsky BA, Senneville É, Abbas ZG, Aragón-Sánchez J, Diggle M, Embil JM, Kono S, Lavery LA, Malone M, van Asten SA, Urbančič-Rovan V, Peters EJG (2020). Guidelines on the diagnosis and treatment of foot infection in persons with diabetes (IWGDF 2019 update). Diabetes Metab Res Rev.

[bib1.bib33] Mijuskovic B, Kuehl R, Widmer AF, Jundt G, Frei R, Gürke L, Wolff T (2018). Culture of Bone Biopsy Specimens Overestimates Rate of Residual Osteomyelitis After Toe or Forefoot Amputation. J Bone Joint Surg Am.

[bib1.bib34] Nadareishvili LHN, Nakaidze N, Nizharadze D, Kutateladze M, Balarjishvili N, Kutter E, Pruidze N (2020). Bacteriophage Therapy as a Potential Management Option for Surgical Wound Infections. PHAGE.

[bib1.bib35] Patel DR, Bhartiya SK, Kumar R, Shukla VK, Nath G (2021). Use of Customized Bacteriophages in the Treatment of Chronic Nonhealing Wounds: A Prospective Study. Int J Low Extrem Wounds.

[bib1.bib36] Peter-Riesch B, Czock A, Uçkay I (2021). Swiss interdisciplinary guidance on good practices for acute and complicated diabetic foot syndromes. Swiss Med Wkly.

[bib1.bib37] Pitocco D, Spanu T, Di Leo M, Vitiello R, Rizzi A, Tartaglione L, Fiori B, Caputo S, Tinelli G, Zaccardi F, Flex A, Galli M, Pontecorvi A, Sanguinetti M (2019). Diabetic foot infections: a comprehensive overview. Eur Rev Med Pharmacol Sci.

[bib1.bib38] Qin CH, Zhou CH, Song HJ, Cheng GY, Zhang HA, Fang J, Tao R (2019). Infected bone resection plus adjuvant antibiotic-impregnated calcium sulfate versus infected bone resection alone in the treatment of diabetic forefoot osteomyelitis. BMC Musculoskelet Disord.

[bib1.bib39] Rossel A, Lebowitz D, Gariani K, Abbas M, Kressmann B, Assal M, Tscholl P, Stafylakis D, Uçkay I (2019). Stopping antibiotics after surgical amputation in diabetic foot and ankle infections-A daily practice cohort. Endocrinol Diabetes Metab.

[bib1.bib40] Saltoglu N, Yemisen M, Ergonul O, Kadanali A, Karagoz G, Batirel A, Ak O, Eraksoy H, Cagatay A, Vatan A, Sengoz G, Pehlivanoglu F, Aslan T, Akkoyunlu Y, Engin D, Ceran N, Erturk B, Mulazimoglu L, Oncul O, Ay H, Sargin F, Ozgunes N, Simsek F, Yildirmak T, Tuna N, Karabay O, Yasar K, Uzun N, Kucukardali Y, Sonmezoglu M, Yilmaz F, Tozalgan U, Ozer S, Ozyazar M (2015). Predictors for limb loss among patient with diabetic foot infections: an observational retrospective multicentric study in Turkey. Clin Microbiol Infect.

[bib1.bib41] Selva Olid A, Solà I, Barajas-Nava LA, Gianneo OD, Bonfill Cosp X, Lipsky BA (2015). Systemic antibiotics for treating diabetic foot infections. Cochrane Database Syst Rev.

[bib1.bib42] Senneville E, Yazdanpanah Y, Cazaubiel M, Cordonnier M, Valette M, Beltrand E, Khazarjian A, Maulin L, Alfandari S, Caillaux M, Dubreuil L, Mouton Y (2001). Rifampicin-ofloxacin oral regimen for the treatment of mild to moderate diabetic foot osteomyelitis. J Antimicrob Chemother.

[bib1.bib43] Senneville E, Joulie D, Blondiaux N, Robineau O (2020). Surgical techniques for Bone Biopsy in Diabetic Foot Infection, and association between results and treatment duration. J Bone Joint Infect.

[bib1.bib44] Tone A, Nguyen S, Devemy F, Topolinski H, Valette M, Cazaubiel M, Fayard A, Beltrand É, Lemaire C, Senneville É (2015). Six-week versus twelve-week antibiotic therapy for nonsurgically treated diabetic foot osteomyelitis: a multicenter open-label controlled randomized study. Diabetes Care.

[bib1.bib45] Uçkay I, Gariani K, Pataky Z, Lipsky BA (2014). Diabetic foot infections: state-of-the-art. Diabetes Obes Metab.

[bib1.bib46] Uçkay I, Aragón-Sánchez J, Lew D, Lipsky BA (2015). Diabetic foot infections: what have we learned in the last 30 years?. Int J Infect Dis.

[bib1.bib47] Uçkay I, Gariani K, Dubois-Ferrière V, Suvà D, Lipsky BA (2016). Diabetic foot infections: recent literature and cornerstones of management. Curr Opin Infect Dis.

[bib1.bib48] Uçkay I, Jornayvaz FR, Lebowitz D, Gastaldi G, Gariani K, Lipsky BA (2018). An Overview on Diabetic Foot Infections, including Issues Related to Associated Pain, Hyperglycemia and Limb Ischemia. Curr Pharm Des.

[bib1.bib49] Uçkay I, Kressmann B, Malacarne S, Toumanova A, Jaafar J, Lew D, Lipsky BA (2018). A randomized, controlled study to investigate the efficacy and safety of a topical gentamicin-collagen sponge in combination with systemic antibiotic therapy in diabetic patients with a moderate or severe foot ulcer infection. BMC Infect Dis.

[bib1.bib50] Uçkay I, Berli M, Sendi P, Lipsky BA (2019). Principles and practice of antibiotic stewardship in the management of diabetic foot infections. Curr Opin Infect Dis.

[bib1.bib51] Uçkay I, Holy D, Schöni M, Waibel FWA, Trache T, Burkhard J, Böni T, Lipsky BA, Berli MC (2021). How good are clinicians in predicting the presence of Pseudomonas spp. in diabetic foot infections? A prospective clinical evaluation. Endocrinol Diabetes Metab.

[bib1.bib52] van Asten SAV, Mithani M, Peters EJG, La Fontaine J, Kim PJ, Lavery LA (2018). Complications during the treatment of diabetic foot osteomyelitis. Diabetes Res Clin Pract.

[bib1.bib53] Waibel FWA, Uçkay I, Sairanen K, Waibel L, Berli MC, Böni T, Gariani K, Lipsky BA (2019). Diabetic calcaneal osteomyelitis. Infez Med.

[bib1.bib54] Waibel F, Berli M, Catanzaro S, Sairanen K, Schöni M, Böni T, Burkhard J, Holy D, Huber T, Bertram M, Läubli K, Frustaci D, Rosskopf A, Botter S, Uçkay I (2020). Optimization of the antibiotic management of diabetic foot infections: protocol for two randomized controlled trials. Trials.

[bib1.bib55] Waibel FW, Schöni M, Kronberger L, Flury A, Berli MC, Lipsky BA, Uçkay I, Jud L (2022). Treatment Failures in Diabetic Foot Osteomyelitis Associated with Concomitant Charcot Arthropathy: The Role of Underlying Arteriopathy. Int J Infect Dis.

[bib1.bib56] Wilson BM, Bessesen MT, Doros G, Brown ST, Saade E, Hermos J, Perez F, Skalweit M, Spellberg B, Bonomo RA (2019). Adjunctive Rifampin Therapy For Diabetic Foot Osteomyelitis in the Veterans Health Administration. JAMA Netw Open.

[bib1.bib57] Wuarin L, Abbas M, Harbarth S, Waibel F, Holy D, Burkhard J, Uçkay I (2019). Changing perioperative prophylaxis during antibiotic therapy and iterative debridement for orthopedic infections?. PLoS One.

[bib1.bib58] Zenelaj B, Bouvet C, Lipsky BA, Uçkay I (2014). Do diabetic foot infections with methicillinresistant Staphylococcus aureus differ from those with other pathogens?. Int J Low Extrem Wounds.

